# N-WASP Is Required for Structural Integrity of the Blood-Testis Barrier

**DOI:** 10.1371/journal.pgen.1004447

**Published:** 2014-06-26

**Authors:** Xiang Xiao, Dolores D. Mruk, Elizabeth I. Tang, R'ada Massarwa, Ka Wai Mok, Nan Li, Chris K. C. Wong, Will M. Lee, Scott B. Snapper, Ben-Zion Shilo, Eyal D. Schejter, C. Yan Cheng

**Affiliations:** 1 The Mary M. Wohlford Laboratory for Male Contraceptive Research, Center for Biomedical Research, Population Council, New York, New York, United States of America; 2 Department of Molecular Genetics, The Weizmann Institute of Science, Rehovot, Israel; 3 Department of Biology, Hong Kong Baptist University, Hong Kong, China; 4 School of Biological Sciences, University of Hong Kong, Hong Kong, China; 5 Harvard Medical School, Boston, Massachusetts, United States of America; University of Nevada School of Medicine, United States of America

## Abstract

During spermatogenesis, the blood-testis barrier (BTB) segregates the adluminal (apical) and basal compartments in the seminiferous epithelium, thereby creating a privileged adluminal environment that allows post-meiotic spermatid development to proceed without interference of the host immune system. A key feature of the BTB is its continuous remodeling within the Sertoli cells, the major somatic component of the seminiferous epithelium. This remodeling is necessary to allow the transport of germ cells towards the seminiferous tubule interior, while maintaining intact barrier properties. Here we demonstrate that the actin nucleation promoting factor Neuronal Wiskott-Aldrich Syndrome Protein (N-WASP) provides an essential function necessary for BTB restructuring, and for maintaining spermatogenesis. Our data suggests that the N-WASP-Arp2/3 actin polymerization machinery generates branched-actin arrays at an advanced stage of BTB remodeling. These arrays are proposed to mediate the restructuring process through endocytic recycling of BTB components. Disruption of N-WASP in Sertoli cells results in major structural abnormalities to the BTB, including mis-localization of critical junctional and cytoskeletal elements, and leads to disruption of barrier function. These impairments result in a complete arrest of spermatogenesis, underscoring the critical involvement of the somatic compartment of the seminiferous tubules in germ cell maturation.

## Author Summary

Mammalian spermatogenesis takes place within a sheltered environment, whereby somatic Sertoli cells protect and guide germ cells as they mature and differentiate. A key structure generated by the protective Sertoli cell epithelium is the blood-testis barrier (BTB), a composite of junctional and cytoskeletal elements, which prevents exposure of post-meiotic spermatids to the immune system. The BTB is a highly dynamic structure, which needs to be dismantled and rapidly rebuilt, in order to allow passage of maturing preleptotene spermatocytes, without compromising their isolation. Here we show that N-WASP, a conserved facilitator of formation of branched actin microfilament arrays, provides a function that is essential for maintenance of an intact BTB. Genetic disruption of N-WASP in mouse Sertoli cells leads to loss of BTB impermeability, resulting in a complete arrest of spermatogenesis at early and post-meiotic stages. Based on the localization patterns of key elements, we propose that branched-actin filaments participate in recycling of BTB materials to ensure the dynamic and efficient maintenance of this structure, one of a series of blood-tissue barriers that preserve privileged organ environments.

## Introduction

Production of sperm in mammals takes place within the seminiferous tubules of the testis. A prominent aspect of this process is a complex series of interactions between the maturing germ cells and the somatic Sertoli cell epithelium, which performs a variety of guidance and protective roles critical for spermatogenic differentiation [1]. A striking example of Sertoli cell support is formation of a blood-testis barrier (BTB) between neighboring Sertoli cells, at the basal aspect of the seminiferous epithelium [2], [3]. The purpose of this barrier is to act as an effective seal between the external environment and the “immune privileged” interior of seminiferous tubules, thereby allowing the maturing germ cells to express necessary antigens, without provoking an autoimmune response.

The BTB is composed of a unique combination of junctional and cytoskeletal structures, making it one of the tightest blood-tissue barriers in the mammalian body [2], [4]. Tight junctions (TJs), gap junctions (GJs), and desmosomes all contribute to the barrier. In addition to these junctional complexes, which can be found in a wide variety of epithelial settings, the BTB harbors unique structures termed ectoplasmic specializations (ES) [5]. Apposing ES are present at the cell bases of both members of neighboring Sertoli-cell pairs, and are composed of highly organized arrays of microfilament bundles, that lie perpendicular to the Sertoli cell plasma membranes, and are sandwiched between the plasma membrane and cisternae of the endoplasmic reticulum [2], [4]. A second, apical ES structure, bearing highly similar ultrastructural features to the basal ES, is found within Sertoli cells at the interface with maturing spermatids, and functions to anchor the spermatids onto the epithelium during spermiogenesis [4]–[6].

A key feature of spermatogenesis is the vectorial journey of maturing spermatocytes, which differentiate from type B spermatogonia residing at the base of the seminiferous epithelium, where the spermatogonial stem-cells are located. With the essential aid of the encompassing Sertoli epithelium, the immotile, inter-connected and differentiating spermatocytes are transported between the Sertoli cells towards the tubule interior. The spermatocytes encounter the BTB as they reach the preleptotene phase of meiosis, at which time they undergo a remarkable “seamless” passage through the barrier [7]. The essence of this process, which takes place during stage VIII of the seminiferous epithelial cycle [8], is a dynamic modification of the BTB, involving dismantling of the existing barrier apical to the preleptotene spermatocytes, closely coupled to formation of a new BTB at a slightly more basal position. Interestingly, this event takes place simultaneously with the release of mature sperm into the tubule lumen at the opposite, luminal edge of the epithelium, suggesting close coordination between restructuring events of the basal and apical ES [9].

We have recently proposed a model for the molecular mechanism underlying spermatogenic transport through the BTB [10], on the basis of the dynamic expression and localization patterns of Arp3, a subunit of the Arp2/3 complex, the primary nucleator of branched-actin arrays in eukaryotic cells. We reported that Arp3 becomes prominent and transiently localizes to the BTB at the time of restructuring, suggesting that Arp2/3-dependent branched-actin arrays form at this critical juncture. These arrays were proposed to promote breakdown of the “old” BTB, and construction of a new barrier via endocytosis-based recycling of existing junctional components. Notably, transient localization of Arp3 is similarly observed at the apical ES, implying that an Arp2/3-dependent mechanism resembling BTB restructuring is employed during dismantling/reformation of this structure as well.

Neuronal Wiskott-Aldrich Syndrome Protein (N-WASP, also known as WASL), the ubiquitous and broadly expressed member of the WAS protein family [11], is one of the major nucleation promoting factors responsible for activation of the microfilament-nucleation capacity of the Arp2/3 complex [12]–[14]. We have previously reported that N-WASP is expressed in Sertoli cells, and that Sertoli-cell-specific knockout of N-WASP results in sterility due to an early arrest in murine spermatogenesis [15]. Here we provide a detailed characterization of the effects of N-WASP disruption in Sertoli cells, thus critically examining and refining the proposed molecular model for BTB restructuring via a genetic approach. Our data implies a requirement for N-WASP in the dynamic remodeling of the BTB during passage of preleptotene spermatocytes at the BTB, and suggests a cascade of events for breakdown of junctional structures and their recycling via activity of Arp2/3-dependent branched-actin arrays.

## Results

### Disruption of N-WASP in Sertoli cells leads to a complete, stage-specific arrest in spermiogenesis

**Figure 1:**
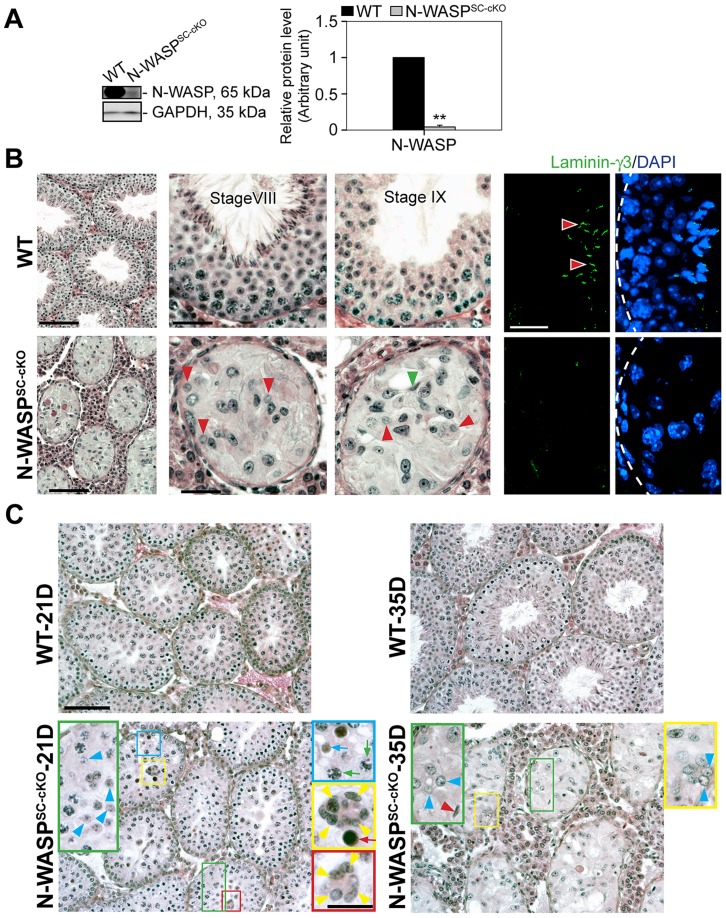
Spermatogenetic arrest and abnormal actin microfilament organization in the seminiferous epithelium of N-WASP^SC-cKO^ mouse testes. (**A**) Lysates (∼50 µg protein) from 8-wk-old testes of N-WASP^SC-cKO^ mice (*Dhh*-Cre; *N-WASP* ^flox^/*N-WASP* ^−^) and age-matched wild-type (WT) control mice (*N-WASP* ^flox^/*NWASP* ^−^) were used for immunoblotting, to assess changes in the expression of N-WASP, with GAPDH serving as a protein loading control. Histograms in this and subsequent figures are composites of quantified immunoblot data (mean ± SD) from *n* = 4 mice, normalized for the loading control. WT protein levels in these graphs were arbitrarily set at 1, against which statistical comparison was performed. **, *P*<0.01. (**B**) **(Left panels)** Hematoxylin and eosin (H&E) staining of paraffin sections, illustrating that specific disruption of N-WASP in Sertoli cells led to major defects in spermiogenesis. N-WASP^SC-cKO^ tubules (bottom) were shrunk in diameter and lacked the normal spermatid-filled seminiferous epithelium seen in control (WT) mouse testes (top). Magnified images of mutant tubules (bottom center and right panels) demonstrate the presence of meiotic round spermatids (red arrowheads) and rare step 11 or 12 spermatids (green arrowhead). Scale bars: 240 µm (left column), and 60 µm (magnified columns). **(Right panels)** Laminin-γ3 chain (green fluorescence), a specific marker of step 13–16 spermatids at the apical ES [20], [21], was not detected in the seminiferous epithelium of N-WASP^SC-cKO^ mouse testes. Absence of advanced-stage spermatids is indicative of a full failure of spermiogenesis in these mutant mice. Scale bar: 50 µm, which applies to other micrographs. (**C**) Histological analysis of age-matched control (WT) and N-WASP^SC-cKO^ tubules from 21- and 35-D (day)-old mice. At 21D and 35D, round spermatids (annotated by blue arrowheads) were found in some tubules from N-WASP^SC-cKO^ testes, illustrating the occurrence of meiosis. However, abnormal multinucleated spermatocytes (annotated by yellow arrowheads) derived from normal spermatocytes (green arrows), were found in 21D N-WASP^SC-cKO^-testes, presumably giving rise to the degenerated spermatocytes (red arrow). Degenerating round spermatids were also noted (blue arrow) in 21D N-WASP^SC-cKO^-testes. In 35D N-WASP^SC-cKO^-testes, elongating spermatids were occasionally found (red arrowhead) and also abnormal multinucleated round spermatids as annotated by blue arrowheads in the yellow boxed area. The lack of elongating spermatids at different stages supports the notion of a full failure of spermiogenesis in N-WASP^SC-cKO^-testes. Scale bars: 120 µm, and 40 µm in insets.

Tissue specific disruption of N-WASP in Sertoli cells (see also [15]) was achieved using a (loxP-based) conditional knock-out (cKO) allele of murine N-WASP [16], and the Sertoli-cell Cre-driver *Desert Hedgehog* (*Dhh*)-Cre [17]–[19]. Notably, N-WASP levels in testes of these N-WASP^SC-cKO^ males were strongly reduced to ∼5% of age-matched controls (**Fig. 1A**), as monitored using specific antibodies (**Table 1**). The residual N-WASP protein is likely to represent germ cell expression [10], which is not affected in this setting. Disrupting N-WASP function in the somatic support cells had a dramatic effect on spermatogenesis. Testes of 8-week-old N-WASP^SC-cKO^ mice weighed ∼20% of age-matched controls. Seminiferous tubule diameter was shrunk by ∼30% and the tubules were virtually devoid of advanced stage germ cells (**Fig. 1B**). Round and elongating spermatids could only be detected in less than 3% of tubules from 8-wk old mice (*n* = 4 mice, a total of 200 randomly selected tubules scored), and more mature (step 13–16) elongating/elongated spermatids were completely absent (**Fig. 1B**), indicating that while meiosis could take place, spermiogenesis failed to go to completion. The histological analysis was further supported and complemented by the absence of laminin-γ3, a specific marker of late spermatids in mouse [20] and rat [21] testes (**Fig. 1B**). It is noteworthy that the spermatogonia/spermatogonial stem-cell (SSC) population is decreased in N-WASP^SC-cKO^ tubules, as assessed using the spermatogonia/SSC marker Utf1 [22], [23] (∼0.2 Utf-1-positive cells/section in mutant testes *versus* ∼1.1 cell/section in wild-type controls), a feature that we ascribe to the overall smaller size of the Sertoli cell population (described below).

**Table 1:** Antibodies used for different experiments in this report.

Antibody	Host species	Vendor	Catalog no.	Application (Dilution)
Actin	Goat	Santa Cruz Biotechnology	sc-1616	IB (1∶200)
Arp3	Mouse	Sigma-Aldrich	A5979	IB (1∶3000), IF (1∶100)
	Rabbit	Proteintech	13822-1-AP	IF (1∶100)
β1-Integrin	Rabbit	Millipore	AB1952	IB (1∶2000), IF (1∶100)
CAR	Rabbit	Santa Cruz Biotechnology	sc-15405	IB (1∶200), IF (1∶100)
Connexin 43	Rabbit	Cell Signaling Technology	3512	IB (1∶200), IHC (1∶100)
Drebrin E	Rabbit	Abcam	ab11068-50	IB (1∶1000)
	Rabbit	Proteintech	10260-1-AP	IF (1∶100)
FAK	Rabbit	Millipore	06-543	IB (1∶1000)
GATA-1	Rat	Santa Cruz Biotechnology	sc-266	IF (1∶50)
p-FAK-Y438 (407)*	Rabbit	Invitrogen	44-650G	IB (1∶1000), IF (1∶100)
p-FAK-Y614 (576)*	Rabbit	Millipore	07-157	IB (1∶1000), IF (1∶100)
GAPDH	Mouse	Abcam	ab8245	IB (1∶1000)
ICAM-2	Rabbit	Santa Cruz Biotechnology	sc-7933	IB (1∶200), IF (1∶100)
Laminin-γ3	Rabbit	Cheng Lab [21]		IF (1∶300)
N-Cadherin	Rabbit	Santa Cruz Biotechnology	sc-7939	IB (1∶200), IF (1∶100)
Nectin-3	Rabbit	Santa Cruz Biotechnology	sc-28637	IB (1∶200)
	Goat	Santa Cruz Biotechnology	sc-14806	IF (1∶50)
N-WASP	Rabbit	Santa Cruz Biotechnology	sc-20770	IB (1∶200), IF (1∶100)
Occludin	Rabbit	Invitrogen	71-1500	IB (1∶250), IF (1∶100)
Utf1	Rabbit	Millipore	AB3383	IF (1∶800)
Goat IgG-Alexa Fluor 488	Donkey	Invitrogen	A11055	IF (1∶250)
Mouse IgG-Alexa Fluor 488	Goat	Invitrogen	A11029	IF (1∶250)
Rabbit IgG-Alexa Fluor 488	Goat	Invitrogen	A11034	IF (1∶250)
Rabbit IgG-Alexa Fluor 555	Goat	Invitrogen	A21429	IF (1∶250)
Biotin-Rat IgG	Goat	Zymed-Invitrogen	62-9540	IF (1∶100)
Streptavidin, FITC conjugated		Pierce	21224	IF(1∶150)

Abbreviations used: IB, immunoblotting; IHC, immunohistochemistry; IF, immunofluorescence analysis.

*, the number bracketed represents the corresponding Tyr residue of p-FAK in the rat testis.

Taken together, these observations suggest a complete failure of spermiogenesis in N-WASP^SC-cKO^ mice, beyond early meiotic phases. To verify this assertion, and rule out the possibility that the observed phenotypes reflect secondary consequences of spermatogenic arrest, we compared the morphologies of seminiferous tubules from younger (21- and 35-day-old) control and mutant mice, closer to the onset of sexual maturity (**Fig. 1C**). A substantial amount of round spermatids were present in N-WASP^SC-cKO^ tubules in 21-day-old mice when meiosis is known to occur, implying that germ cells in these tubules were capable of entering meiosis, and properly initiated spermiogenesis. However, degeneration at both spermatid and spermatocyte stages is readily apparent, such as the presence multi-nucleated round spermatids and multi-nucleated spermatocytes by 21 and 35 dpp *versus* 8-wk old mutants (**Fig. 1C** **** ***vs.*** **1B**), implying a block to maturation at these stages of spermiogenesis.

### Disorganization of the Sertoli-cell actin cytoskeleton and functional impairment of the BTB in N-WASP^SC-cKO^ tubules

**Figure 2:**
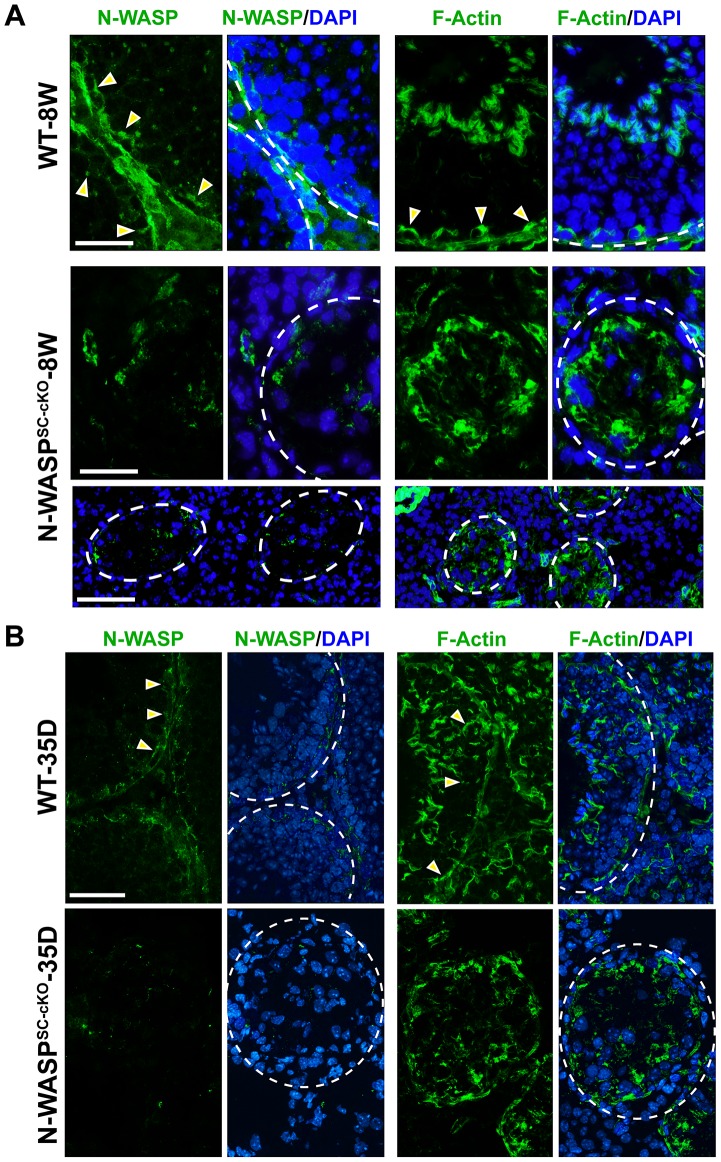
Sertoli cell-specific knockout of N-WASP leads to disorganization of F-actin in the seminiferous epithelium. (**A**) Immunofluorescence analysis of sectioned seminiferous tubules from 8W (week)-old control (WT) and N-WASP^SC-cKO^ testes, showing the expression and localization patterns of N-WASP (green fluorescence, left) and F-actin (FITC-phalloidin, green fluorescence, right). Yellow arrowheads in the control panels point to the localization of either N-WASP or F-actin at the site of the BTB near the basement membrane of the tunica propria, which is annotated by a broken white-line. Cell nuclei were visualized by DAPI staining. Scale bars: 80 µm in the first two rows, and 240 µm in the third row. (**B**) A similar analysis was performed on testes from younger, 35D (day)-old mice. Actin microfilament organization in N-WASP^SC-cKO^ tubules was strongly disrupted at this stage as well. Scale bar: 80 µm.

We next turned to a detailed analysis of Sertoli cell features in N-WASP^SC-cKO^ mice in order to understand the basis for the dramatic impact on spermiogenesis. We had previously demonstrated that N-WASP^SC-cKO^ mice possess an outwardly intact Sertoli cell epithelium (15). This was now supported by assessing the size of the Sertoli cell population in sectioned tubules using GATA-1, a nuclear Sertoli cell marker [24]. The total number of Sertoli cells within the epithelium is somewhat smaller in mutant tubules (∼4 Sertoli cells/cross-section) in comparison to wild-type controls (∼8 Sertoli cells/cross-section) due to shrinkage in tubule diameter. Similar population-size values were obtained, however, regardless of age, consistent with a generally healthy epithelium that does not succumb to cell death over time. Examination of microfilament distribution via staining with fluorescent FITC-phalloidin suggested, however, major disturbances to Sertoli cell cytoplasmic organization (**Fig. 2A, B**). The F-actin network in the seminiferous epithelium of 8 week-old N-WASP^SC-cKO^ mice was considerably disrupted, as F-actin was no longer confined to the basal ES at the BTB, but was instead diffusely localized throughout the basal compartment (**Fig. 2A**). A similar lack of organization was observed in tubules from younger (35 day old) mutant mice (**Fig. 2B**), implying that, similar to the effects on the germline, the somatic phenotypes do not result from tissue deterioration over time, but rather reflect fundamental and persistent defects in spermatogenesis.

**Figure 3:**
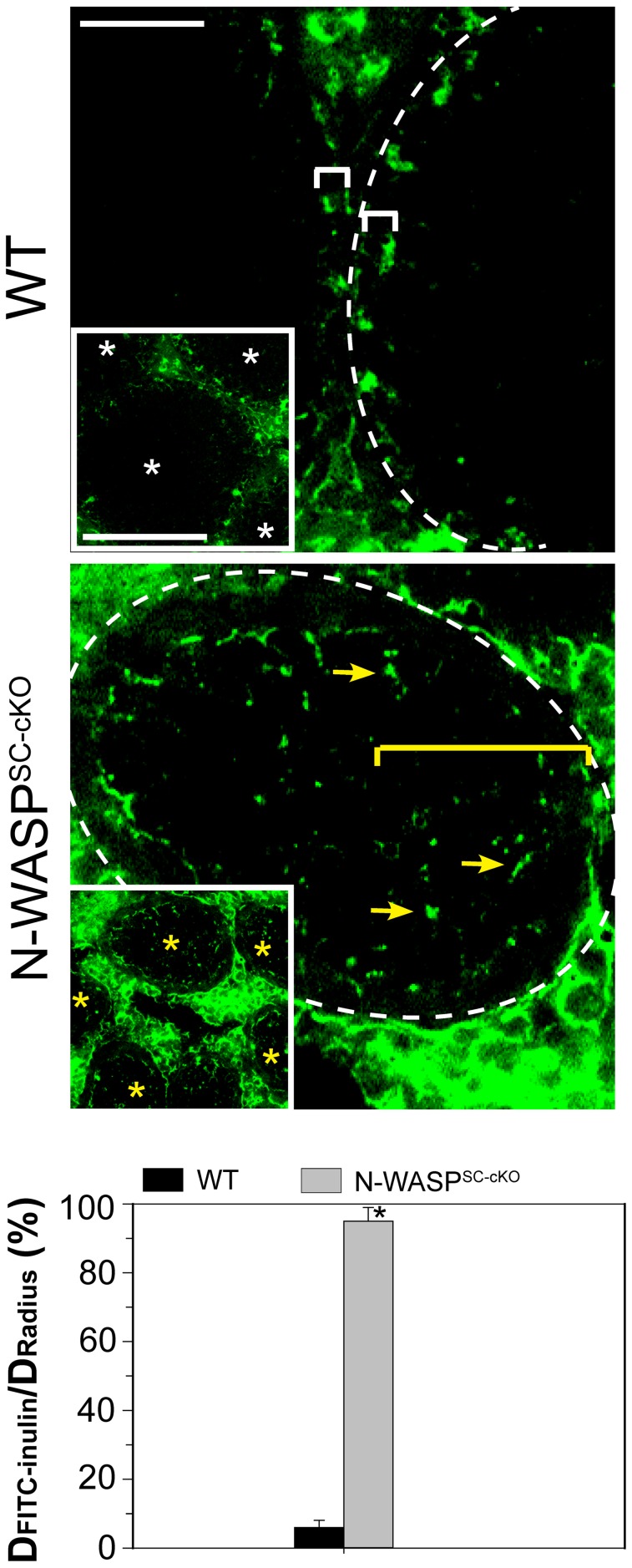
BTB integrity is compromised in testes of N-WASP^SC-cKO^ mice. Images show localization within seminiferous tubules of FITC-inulin (green), a small fluorescent molecule injected into the blood system of control (WT) and N-WASP^SC-cKO^ mice (see *Materials and Methods* for detailed experimental protocol). In control mouse testes, the functional BTB blocked FITC-inulin from entering into the apical (adluminal) compartment, and the distance traveled by the marker was limited to the basal compartment (annotated by a white bracket). The lower magnification inset shows four seminiferous tubules (white asterisks) that are devoid of green fluorescence inside the epithelium. In N-WASP^SC-cKO^ mouse tubules, the marker penetrated deep inside the seminiferous epithelium (yellow arrows), reaching the tubule lumen (annotated by yellow bracket). The lower magnification inset shows five such tubules (yellow asterisks), demonstrating that disruption of BTB integrity is a common feature of N-WASP^SC-cKO^ mouse testes. Scale bars: 50 µm (magnified image), and 200 µm in insets. Data from BTB integrity assays were semi-quantified and are shown in the bar graph, which displays the distance traveled by FITC-inulin *vs.* the tubule radius for *n* = 5 mice (8–14 weeks old) in each group. *, *P*<0.01.

ES structures constitute the primary site of microfilament concentration within Sertoli cells. The highly disorganized nature of microfilament distribution within the N-WASP^SC-cKO^ seminiferous epithelium suggested therefore that ES-related structures such as the BTB may be affected in the mutants. Furthermore, N-WASP is an established promoting factor of Arp2/3-based branched actin nucleation, and our recent analysis of the expression and localization patterns of Arp3, a subunit of the Arp2/3 complex, led us to propose a model for the involvement of this primary nucleator of branched actin arrays in the cyclical dismantling and restructuring of the BTB [10]. We therefore sought to examine whether the barrier function of the BTB is compromised following Sertoli-cell disruption of N-WASP. Towards this end we employed an *in vivo* BTB integrity assay, which is based on the capacity of a functional BTB to block passage of a fluorescent marker (FITC-inulin) from basal to adluminal tubule compartments [25], [26]. In control adult mice (*n* = 5 mice), FITC-inulin failed to penetrate the BTB, which lies close to the basement membrane, and no fluorescence was found inside the adluminal compartment (**Fig. 3**). In contrast, FITC-inulin readily penetrated into the adluminal compartment in the tubules of age-matched N-WASP^SC-cKO^ mouse testes (*n* = 5 mice), and could be detected as far as the tubule lumen (**Fig. 3**), implying a breakdown of the BTB.

### Widespread abnormalities of BTB junctional components in N-WASP^SC-cKO^ seminiferous tubules

**Figure 4:**
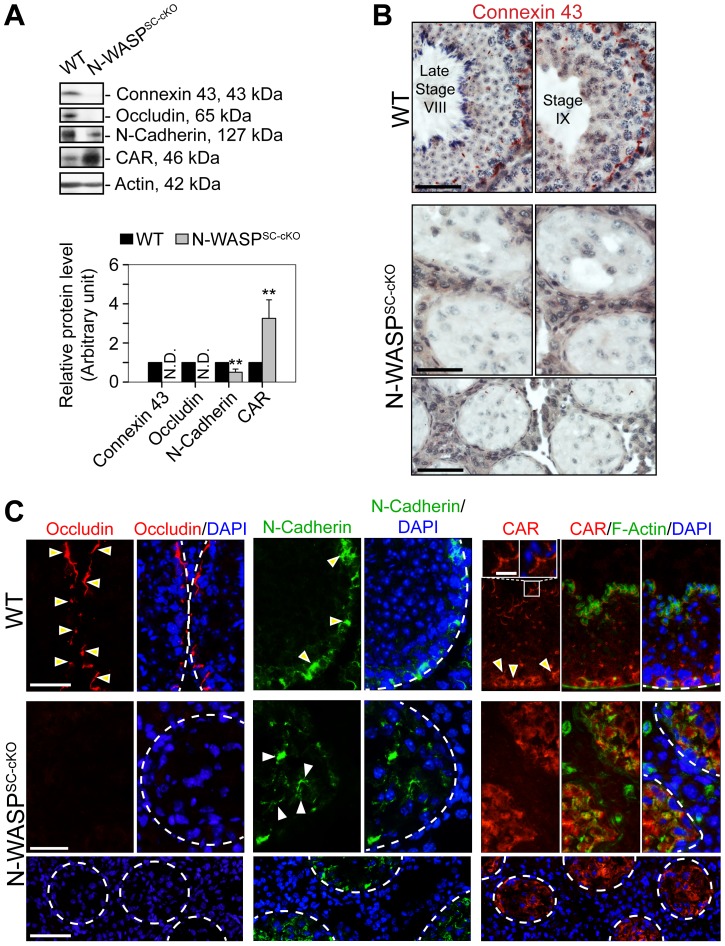
BTB junctional and basal ES proteins are absent or abnormally localized in the seminiferous epithelium of N-WASP^SC-cKO^ mouse testes. (**A**) Immunoblotting data using lysates of testes from N-WASP^SC-cKO^ and age-matched control mice, to examine changes in the expression of integral membrane proteins of GJs (connexin 43), TJs (occludin, CAR), and the basal ES (N-cadherin). β-Actin served as a protein loading control. Histogram generated as in Figure 1. **, *P*<0.01. N.D., not detectable. (**B**) Immunohistochemical localization of connexin 43 in the seminiferous epithelium of mouse testes. In WT testes, connexin 43 appears as a reddish brown precipitate, prominently expressed near the BTB, as well as at the apical ES (near lumen) at early stage VIII. Connexin 43 was virtually undetectable in the N-WASP^SC-cKO^ epithelium, consistent with the immunoblot analysis shown in (**A**). Scale bars, 80 µm in the first two rows; 240 µm in the third row. (**C**) Immunofluorescence analysis of occludin (red fluorescence, left), N-cadherin (red fluorescence, middle) and CAR (red fluorescence, right) in the seminiferous epithelium of N-WASP^SC-cKO^ and WT control mice. Yellow arrowheads indicate the localization of occludin, N-cadherin or CAR at the BTB, which is located near the basement membrane at the tunica propria, annotated by a white broken-line. In the micrographs illustrating localization of CAR (right), the boxed area was magnified and shown in insets, illustrating that CAR was also associated with the apical ES at the elongating/elongated spermatid/Sertoli cell interface. Cell nuclei were visualized by DAPI staining. Scale bars: 80 µm in the first two rows, 240 µm in the third row, and 15 µm in insets.

The observed impairment of BTB barrier function led us to examine possible structural defects in this complex system, by assessing the localization patterns of major constituent proteins. Connexin-43, a transmembrane protein, is an established structural and functional constituent of Sertoli cell gap junctions [27], [28], while occludin, an integral member of intercellular TJs, including the TJ portion of the BTB, acts to stabilize TJs and to optimize their function as barriers [29]–[31]. Remarkably, both connexin-43 and occludin were virtually undetectable in N-WASP^SC-cKO^ tubules (**Fig. 4A,C**), implying a major disruption of the tight and gap junction components of the N-WASP^SC-cKO^ BTB.

Given the prominent effects observed, we extended our analysis to include two additional transmembrane BTB elements, the basal ES component N-cadherin, an integral constituent of the BTB, and the TJ integral membrane protein coxsackievirus and adenovirus receptor (CAR) [32], [33]. In contrast to the tight basal localization normally observed for these proteins, their distribution in the seminiferous epithelium of N-WASP^SC-cKO^ tubules was no longer restricted, and they were detected throughout the Sertoli cell epithelium (**Fig. 4A,C**), further strengthening the notion of an impaired BTB, supporting the functional data shown in **Fig. 3**.

**Figure 5:**
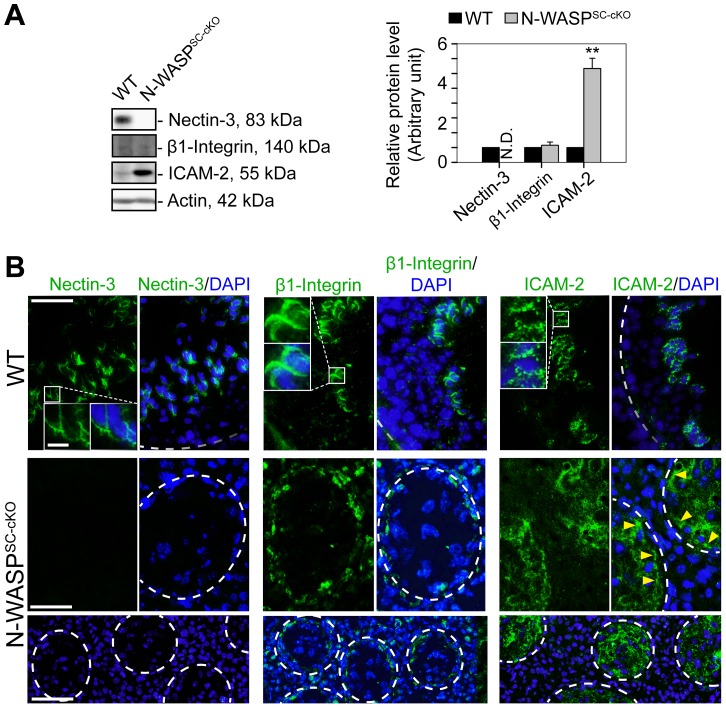
Abnormal localization and expression patterns of apical ES proteins in the seminiferous epithelium of N-WASP^SC-cKO^ mouse testes. (**A**) Immunoblotting data using lysates of testes from N-WASP^SC-cKO^ and age-matched control mice, to examine changes in the expression of the apical ES integral membrane proteins nectin-3, β1-integrin, and ICAM-2. β-Actin served as a protein loading control. Histogram generated as in Figure 1. **, *P*<0.01. N.D., not detectable. (**B**) Immunofluorescence analysis of nectin-3 (green fluorescence, left), β1-integrin (green fluorescence, middle) and ICAM-2 (green fluorescence, right) in the seminiferous epithelium of control (WT) and N-WASP^SC-cKO^ mice. Boxed areas are magnified and shown in insets. Nectin-3, β1-integrin and ICAM-2 were associated with apical ES in control (WT) testes. In N-WASP^SC-cKO^ mouse testes, nectin-3 was considerably diminished to a level almost undetectable in the seminiferous epithelium, consistent with the immunoblotting data shown in (**A**). While β1-integrin and ICAM-2 were detectable in the mutant testes, they were mis-localized and were both found near the disrupted BTB (see yellow arrowheads that annotate ICAM-2 in the mutant testes). Relative location of the basement membrane is annotated by a white broken-line). Cell nuclei were visualized by DAPI staining. Scale bars: 80 µm in the first two rows, 240 µm in the third row, and 15 µm in insets.

Both the general disruption of microfilament organization and the relatively early arrest in the progress of spermatogenesis suggest that the second Sertoli cell ES structure, the apical ES, would not form properly - if at all - in the N-WASP^SC-cKO^ seminiferous epithelium. We examined this issue directly by assessing the localization patterns of representative apical ES components. Nectin-3, a spermatid-specific adhesion molecule that mediates Sertoli cell-spermatid attachment at the apical ES [34], was undetectable in the mutant tubules (**Fig. 5A,B**), consistent with the arrest of spermiogenesis at immature, early spermatid stages. Furthermore, both β1-integrin, a Sertoli cell-specific adhesion protein [35], [36], and ICAM-2, an adhesion protein normally found in both Sertoli cells and spermatids [37], were mis-localized in N-WASP^SC-cKO^ Sertoli cells, and no longer restricted to the adluminal compartment (**Fig. 5A,B**). Taken together, these observations confirm the expectation of a severely disrupted apical ES.

### BTB restructuring in N-WASP^SC-cKO^ seminiferous tubules arrests at the phase requiring branched-actin nucleation

**Figure 6:**
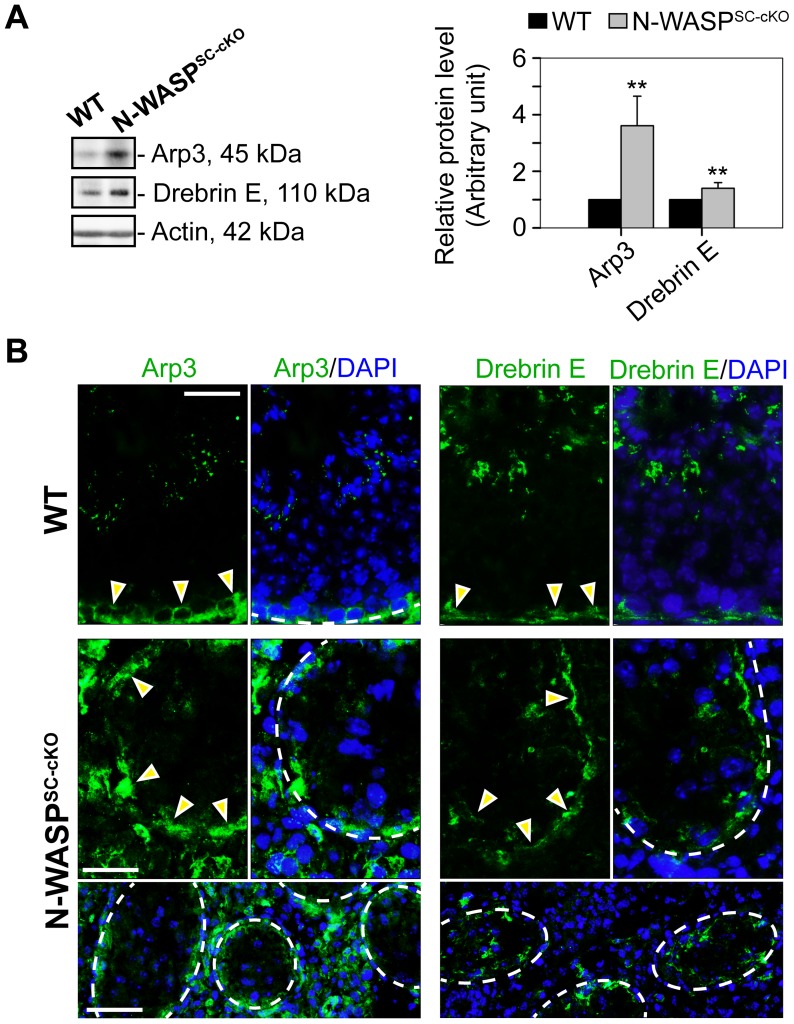
The branched microfilament regulators Arp3 and drebrin E are properly localized to the BTB in N-WASP^SC-cKO^ tubules. (**A**) Immunoblotting data using lysates of testes from N-WASP^SC-cKO^ and age-matched control mice demonstrate up-regulation in Arp3 and drebrin E expression in mutant mouse testes *vs.* the age-matched control testes with β-actin served as a protein loading control. Histogram generated as in Figure 1. **, *P*<0.01. (**B**) Immunofluorescence analysis of Arp3 (green fluorescence, left) and drebrin E (green fluorescence, right) in the seminiferous epithelium of control (WT) and N-WASP^SC-cKO^ mice. Yellow arrowheads mark the localization of either Arp3 or drebrin E at the BTB, which is near the basement membrane of the tunica propria (annotated by a broken white-line). Unlike basal ES proteins (see Figure 4) and apical ES proteins (see Figure 5), which were mis-localized in the seminiferous epithelium of mutant testes, these branched actin regulatory proteins remained properly localized to the damaged BTB. Cell nuclei were visualized by DAPI staining. Scale bars: 80 µm in the first two rows, and 240 µm in the third row.

In contrast to the widespread disturbances to junctional and cytoskeletal BTB components in the N-WASP^SC-cKO^ seminiferous epithelium, we found that two elements of the branched-actin nucleation machinery, namely Arp3 and drebrin E, were properly localized and robustly expressed (**Fig. 6A, B**). In particular, the Arp3 subunit of the Arp2/3 complex, which normally localizes to the BTB vicinity during the restructuring phase of stage VIII, displayed a tightly restricted localization to the basal ES in the seminiferous epithelium of mutant mice, analogous to the age-matched control mouse testis (**Fig. 6A, B**). We have recently demonstrated that drebrin E, the non-neuronal isoform of the actin-binding element drebrin [38], acts to recruit Arp3 to the BTB [39]. Examination of N-WASP^SC-cKO^ tubules revealed that, like Arp3, drebrin E retained its basally restricted expression pattern, similar to WT control mouse testes (**Fig. 6B**). Taken together, the defects in BTB structure, coupled with the observed basal localization of Arp3 and its recruiting partner drebrin E, suggest a molecular scenario in which dismantling of the BTB is properly initiated in the seminiferous epithelium of N-WASP^SC-cKO^ mouse testes during the epithelial cycle, but is arrested at a relatively late stage of the process, when the Arp2/3 actin-nucleation promoting activity of N-WASP must come into play, in order to enable recycling of “old” BTB components and formation of a “new” barrier.

### Disruption of spatiotemporal expression of p-FAK-Tyr^438^ and -Tyr^614^ in the seminiferous epithelium of N-WASP^SC-cKO^ testes

**Figure 7:**
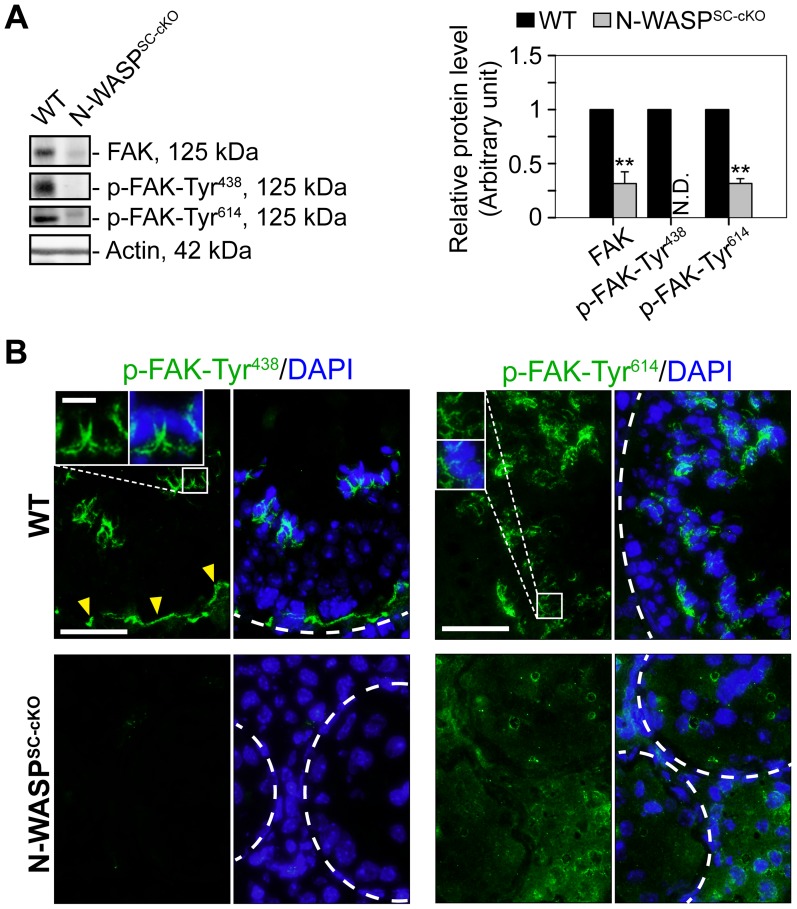
Modifications in the localization and expression patterns of the ES regulatory protein FAK and its phosphorylated forms in N-WASP^SC-cKO^ tubules. (**A**) Immunoblotting data using lysates of testes from N-WASP^SC-cKO^ and age-matched control mice, to examine changes in the expression of the non-receptor protein tyrosine kinase FAK and its phosphorylated/activated forms p-FAK-Tyr^438^ and -Tyr^614^. β-Actin served as a protein loading control. Histogram generated as in Figure 1. **, *P*<0.01. N.D., not detectable. (**B**) Immunofluorescence analysis of p-FAK-Tyr^438^ (green fluorescence, top) and p-FAK-Tyr^614^ (green fluorescence, bottom), in the seminiferous epithelium of mouse testes in both animal groups. Yellow arrowheads annotate p-FAK-Tyr^438^ that was normally localized to the BTB, which was located near the basement membrane at the tunica propria (annotated by white broken-line). Unlike p-FAK-Tyr^438^ which was localized both at the BTB and the apical ES, p-FAK-Tyr^614^ was restrictively expressed at the apical ES, analogous to p-FAK-Tyr^576^ in the rat testis [42]. Boxed areas were magnified and shown in insets, illustrating that p-FAK-Tyr^438^ and -Tyr^614^ were normally associated with the apical ES at the elongating/elongated spermatid/Sertoli cell interface. The expression of p-FAK-Tyr^438^ was considerably diminished in the seminiferous epithelium of N-WASP^SC-cKO^ mouse testes, consistent with the immunoblot data shown in (**A**). While the expression p-FAK-Tyr^614^ was considerably down-regulated, yet it remained detectable in the seminiferous epithelium of N-WASP^SC-cKO^ mouse testes, and its spatiotemporal expression was considerably altered. For instance, p-FAK-Tyr^614^ was no longer restricted to the apical ES at the elongating/elongated spermatids but associated with round spermatids and spermatocytes and also at the damaged BTB. Cell nuclei were visualized by DAPI staining. Scale bars: 80 µm, and 15 µm in insets.

Studies in the rat testis have shown that the non-receptor protein tyrosine kinase FAK, a regulator of cell adhesion in a wide variety of cellular contexts [40], functions to coordinate events at the basal and apical ES. Key features of FAK activity in the seminiferous epithelium are the restricted spatiotemporal patterns of the phosphorylated forms p-FAK-Tyr^407^ (i.e., p-FAK-Tyr^438^ in mouse testes) [41] and p-FAK-Tyr^576^ (i.e., p-FAK-Tyr^614^) [42]. The implication is that p-FAK-Tyr^407^ serves as a molecular switch regulating BTB adhesion capacities, via changes in actin polymerization kinetics [41], [43]. We therefore used general and form specific antibodies to assess FAK (**Table 1**) expression and localization patterns in the tubules of N-WASP^SC-cKO^ mice. Expression levels of the p-FAK-Tyr^614^ form were significantly lower, while the key phosphorylated form p-FAK-Tyr^438^ was practically undetectable via either immunoblot or immunofluorescence microscopy (**Fig. 7A–C**). Absence of Tyr-phosphorylated FAK proteins from the BTB microenvironment thus considerably impaired the adhesive capacity of this key ES structure, and this notion was also supported by data shown in **Fig. 3**.

## Discussion

Blood-tissue barriers are designed to protect sensitive organ environments from infiltration by harmful substances. Efficient barrier properties need to be coupled, however, with a degree of permeability, and a capacity for selective passage of factors essential for tissue differentiation and function. The dynamic behavior of the BTB, which governs the initial phases of spermatogenesis in the seminiferous tubules of the testis, provides a striking example of such situations. In this instance, the BTB is required to act as an efficient barrier, while enabling passage not only of molecular elements, but also of entire cells, namely, the differentiating preleptotene spermatocytes. This is made possible by a tissue remodeling process, involving near simultaneous dismantling and construction of the barrier at positions ahead and in the wake of transiting spermatocytes, making their way towards the adluminal compartment of the seminiferous epithelium.

In the current study, we demonstrate that the nucleation promoting factor N-WASP is required in murine Sertoli cells for maintenance of a properly structured BTB. Sertoli cell-specific disruption of N-WASP results in abnormal spatiotemporal expression patterns of key BTB elements, and in loss of barrier impermeability, suggesting major impairment of BTB structure and function. Key constituents of both the tight junction (occludin, CAR) and gap junction (connexin-43) components of the BTB are either significantly mis-localized or absent altogether. Importantly, major structural abnormalities are apparent in organization of the basal ES, the unique, hallmark component of the BTB. In particular, the tightly packed arrangement of basal ES microfilaments is disrupted, and this key cytoskeletal structure becomes dispersed in the Sertoli cell cytoplasm. Similar dispersal is observed for N-cadherin, an adherens junction component that normally displays tight association with the basal ES. Impairment of the adhesive properties of the BTB are further implied by the marked reduction in levels of phospho-FAK proteins, key regulators of ES-based adhesion. Taken together, these data present a substantial list of defects in BTB structure and functional properties, demonstrating that disruption of N-WASP in Sertoli cells results in significant deterioration of BTB barrier capabilities.

The generally disorganized spatial distribution of junctional and cytoskeletal BTB components in N-WASP^SC-cKO^ tubules is in marked contrast to elements of the branched-actin nucleation system, which retain their proper spatiotemporal localization patterns. Both Arp3, a subunit of the Arp2/3 complex, and drebrin E, a factor that mediates Arp3 localization in Sertoli cells, preserve their tight basal localization during stage VIII of the seminiferous epithelial cycle in the mutant tubules. These observations are significant for several reasons. First, they argue that abnormal localization patterns do not result from a general disruption of cytoplasmic organization in N-WASP^SC-cKO^ tubules, but are likely to arise from specific defects caused by the absence of actin nucleation. This assertion is also supported by the morphological similarities between N-WASP^SC-cKO^ tubules at different ages, implying that mutant phenotypes are a primary consequence of disrupting N-WASP function, and not a result of tissue degeneration over time. Second, our observations identify a particular juncture during which lack of N-WASP function becomes apparent. A timeline consistent with the various observations suggests proper localization of the Arp2/3 complex (mediated in part by drebrin E) to the basal aspect of Sertoli cells, at the stage when BTB restructuring is required. Under normal circumstances, N-WASP then acts to stimulate Arp2/3 and promote nucleation of branched actin arrays necessary for BTB restructuring. The actual involvement of the N-WASP-Arp2/3 machinery is likely to take place after restructuring is underway, and when many of the BTB components are transiently displaced, to allow the dynamic events of the process to unfold. Arrest of restructuring in the N-WASP^SC-cKO^ mutant tubules occurs therefore at a critical phase, when the BTB has been dismantled, but is now incapable of forming a new intact barrier, such as at the rear end of the preleptotene spermatocytes under transport at the immunological barrier.

**Figure 8:**
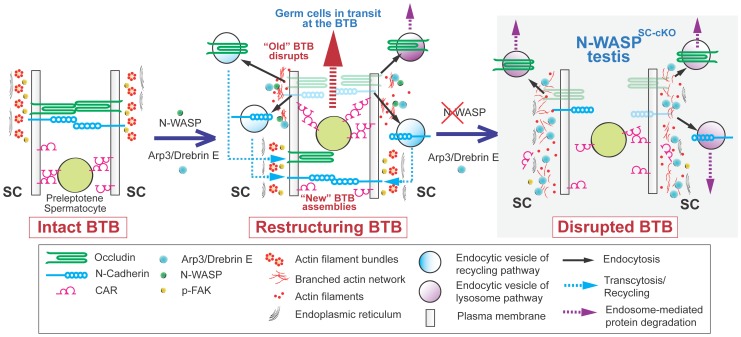
A model illustrating the suggested roles of N-WASP and branched-actin polymerization in BTB dynamics. (Left) The BTB, composed of junctional and cytoskeletal structural and regulatory elements, provides a seal between the basal (down) and adluminal (up) compartments of the epithelium in seminiferous tubules. (Middle) At stage VIII of the epithelial cycle, the BTB undergoes restructuring, involving near simultaneous dismantling of the “old” BTB via endocytosis, to accommodate the apically directed transport of preleptotene spermatocytes between neighboring Sertoli cells, and assembly of a “new” BTB, to maintain the barrier. These events are mediated by the branched actin regulators drebrin E and Arp3, which localize to the BTB, and the nucleation promoting factor N-WASP, which activates branched-actin polymerization. The proposed role for the branched-actin arrays is to facilitate endocytic vesicle-mediated protein trafficking, so that integral membrane proteins at the “old” BTB can be recycled to the basal region and contribute to assembly of a “new” BTB. (Right) In N-WASP^SC-cKO^ testes lacking Sertoli-cell N-WASP function, the branched-actin polymerization machinery can no longer mediate proper endocytic vesicle-mediated protein recycling, and the BTB, once dismantled, cannot be regenerated.

Identification of the juncture at which the branched actin polymerization system contributes to BTB remodeling allows for informed speculation regarding the underlying molecular mechanism. The highly dynamic nature of the restructuring process, the involvement of multiple components, and the availability of “building blocks” for BTB formation following dismantling of the “old” structure all suggest that recycling of existing components should play a prominent role in “new” BTB formation. An involvement of Arp2/3-based branched actin polymerization in endocytic recycling of proteins and membrane has been demonstrated in a wide variety of cellular systems [44]–[46]. Based on these precedents and on our analysis of defective spermatogenesis in N-WASP^SC-cKO^ seminiferous tubules, we propose the following model for branched actin mediation of BTB restructuring (**Figure 8**). By stage VIII of the epithelial cycle, developing spermatocytes, derived from spermatogonial stem cells and being transported between Sertoli cells, have progressed to the preleptotene phase of meiosis, and encounter the BTB. In order to allow passage of the germ cells towards the tubule interior, dismantling of the barrier is initiated, via dispersal of basal ES microfilaments and endocytosis of junctional components. The Arp2/3 complex is recruited by drebrin E to the BTB at this time. N-WASP stimulation of Arp2/3 nucleation activity leads to formation of branched actin arrays on the endocytic vesicles, which are now directed basally, to deposit their cargo (e.g., tight and gap junction proteins) at the site of new BTB construction. In the absence of branched actin functionality, such as in the case of N-WASP disruption in Sertoli cells, vesicles containing “old” BTB elements are not properly directed and either become mis-localized, or diverted towards lysosomal degradation.

Key open questions raised by this model include the nature of the signal converting N-WASP to its active form in this context, the cues by which recycling endosomes are targeted to a particular membrane domain for new BTB formation, and the reason(s) why spermatocytes fail to mature properly following BTB failure. The latter issue may be a reflection of additional impairments to Sertoli cell function, as these cells guide and nurture sperm differentiation throughout the process. One aspect to consider in this context is the involvement of branched actin arrays in the formation of the apical ES, which displays many structural similarities to the ES portion of the BTB, and acts to anchor mature spermatids to the apical end of the Sertoli-cell epithelium just prior to their release into the tubule lumen. The relatively early arrest of spermatogenesis in N-WASP^SC-cKO^ tubules precludes the ability to properly address this issue following complete elimination of N-WASP activity in Sertoli cells. However, we previously demonstrated [10] that partial interference with N-WASP function, following application of the inhibitor wiskostatin, results in mis-orientation of spermatid heads, while the BTB remains intact, suggesting defects in the anchoring capacity of the apical ES.

In conclusion, we describe here an essential requirement for the molecular machinery promoting polymerization of branched actin arrays in the events that mediate dynamic formation of the BTB, and hence, in the proper progress of mammalian spermatogenesis, particularly spermiogenesis. Our findings suggest that this process employs a complex interplay between junctional remodeling, vesicular trafficking and dynamic organization of the actin cytoskeleton, to ensure differentiation while maintaining the integrity of a key blood-tissue barrier.

## Materials and Methods

### Mouse genetics

The use of animals for the studies reported herein was approved by The Rockefeller University Institutional Animal Care and Use Committee with Protocol Number 12-506. *N-WASP* was specifically deleted in Sertoli cells of the mouse testis as previously described [15]. In brief, *Dhh*-Cre bearing mice [47] were crossed to heterozygotes for the null allele *N-WASP* ^−^ [48] to obtain *Dhh*-Cre/+; N-WASP^−/+^ progeny. These F1 mice were then crossed to mice homozygous for the conditional KO allele N-WASP^flox^ [16], and *Dhh*-Cre; *N-WASP* ^flox^/*N-WASP* ^−^ males (*i.e.*, N-WASP^SC-cKO^ male mice) were identified among the progeny. Mice at age 21- and 35-day postpartum (dpp), as well as at 8-, 9-, and 11-week (wk) of age were used. Age-matched wild type (WT) *N-WASP* ^flox^/*N-WASP* ^−^ mice served as the corresponding control mice. Data shown herein are representative findings from single experiments, but each experiment was repeated with at least *n* = 3–5 mutant mice and the corresponding number of WT controls, and yielded similar results.

### Morphometric analysis

Morphometric analysis was performed to score Utf1- or GATA-1-positive cells in the seminiferous epithelium of mice from *Dhh*-Cre; *N-WASP* ^flox^/*N-WASP* ^−^ males (*i.e.*, N-WASP^SC-cKO^ male mice) *versus* aged-matched *N-WASP* ^flox^/*N-WASP* ^−^ WT (control) mice, as earlier described [23], [26]. In brief, serial frozen sections (7 µm) of testes from these mice were obtained in a cryostat at −21°C. During serial sectioning, every fifth section was removed and collected on microscopic slide, and a total of 5 sections was collected from each testis from both groups. Data presented were representative results from a total of 15 cross sections of testes from 3 mice. Utf1- or GATA1-positive cells were scored from randomly selected 30 tubules per section of testis (i.e., about 150 tubules per testis and a total of 450 tubules in each mouse group) using the 10× Objective of an Olympus BX 61 fluorescence microscope. GATA1 (globin transcription factor 1) is a Sertoli cell-specific marker in the seminiferous epithelium [24], whereas Utf1 is a marker of spermatogonia/SSC specific to A_s_ (A_single_), A_pr_ (A_paired_) and short chain of A_al_ (A_alinged_) spermatogonia including SSC [22]. Positive Utf1 or GATA1 stained cells were further verified using the 40× Objective to ensure that the fluorescence staining was not an artifact. For GATA1, the number of Sertoli cells in the epithelium was estimated from both 35-day and 8-wk old mice., while for Utf1-positive cells, only 8-wk-old mice were used. Only round-shaped and not cross sections were used for scoring.

### BTB integrity assay

To assess if the disruption of N-WASP in Sertoli cells would impede BTB integrity *in vivo* in mice, a functional assay was performed, as earlier described [25], [26]. This assay is based on the ability of an intact BTB to block the diffusion of a small fluorescence tag, FITC-conjugated inulin (Mr 4.6 kDa) (Sigma-Aldrich) from the basal to the apical compartment of the seminiferous epithelium. Adult mice from 9–14 wk of age (*n* = 5 control (WT) mice *vs. n* = 5 N-WASP^SC-cKO^ mice) were under anesthesia with ketamine HCl (∼100 mg/kg b.w.) together with an analgesic xylazine (∼10 mg/kg b.w.), administered intramuscularly (i.m.). A small incision, about 0.5-cm, was made in the skin over the jugular vein to expose the blood vessel, and ∼1 mg FITC-inulin dissolved in ∼75 µl PBS (10 mM sodium phosphate, 0.15 M NaCl, pH 7.4 at 22°C) was administered into the jugular vein via a 28-gauge needle. About 45 min thereafter, mice were euthanized by CO_2_ asphyxiation by slow (20–30% per minute) displacement of chamber air with compressed CO_2_ for ∼12 min. Testes were removed, snap-frozen in liquid nitrogen, and embedded in Tissue-Tek O.C.T. (optimal cutting temperature) compound (Sakura). Frozen sections (10 µm) were obtained in a cryostat at −21°C. Fluorescence images were obtained using an Olympus BX61 Fluorescence Microscope. Mice from both control (WT) and conditional KO groups were processed in the same experimental session for comparison to avoid inter-experimental variations. To yield semi-quantitative information regarding the BTB integrity, distance (D) traveled by the fluorescence tag (D_FITC-inulin_) from the basement membrane in a seminiferous tubule *versus* radius of the tubule (D_Radius_) in N-WASP^SC-cKO^ testes was compared to WT control mice. For oblique sections of tubules, D_Radius_ was obtained by averaging the shortest and the longest distance from the basement membrane. About 30 tubules were randomly scored from each mouse testis and a total of 5 mice from each group was scored and compared.

### Immunoblotting

Immunoblotting was performed using lysates from 3–4 mouse testes, as earlier described [49]. To obtain sample lysates, tissues from snap-frozen mouse testes stored at −80°C were homogenized and sonicated in ice-cold IP lysis buffer [10 mM Tris, 0.15 M NaCl, 1% NP-40, and 10% glycerol (v/v), pH 7.4 at 22°C, freshly supplemented with protease and phosphatase inhibitor cocktails (Sigma-Aldrich) at a 1∶100 dilution (v/v), as instructed by the manufacturer]. Total protein concentration was estimated by using the detergent-compatible *DC*™ Protein Assay kit (Bio-Rad), using BSA as a standard. About 30–50 µg of protein lysate from each sample was used for immunoblotting. Immunoblots were visualized by enhanced chemiluminescence (ECL), using a kit prepared as described [50]. Gel images were obtained using a Fujifilm LAS-4000 Mini imaging system with Multi Gauge software (Version 3.1; Fujifilm), and immunoblots were then analyzed by using Scion Image (Version 4.0.3.2; Scion Corporation; http://mesonpi.cat.cbpf.br/e2002/cursos/NotasAula/ScnImage.pdf). Data were normalized against β-actin, which served as a protein loading control.

### Immunofluorescence microscopy

Immunofluorescence microscopy was performed as described earlier [51] using corresponding antibodies (**Table 1**). Cryosections (7-µm-thick) obtained in a cryostat at −21°C from snap-frozen mouse testes, were fixed at room temperature either in Bouin's fixative for 5 min or in 4% paraformaldehyde (PFA) (w/v) dissolved in PBS (10 mM NaH_2_PO_4_ and 0.15 M NaCl, pH 7.4 at 22°C) for 10 min. Sections were permeabilized in 0.1% Triton X-100 (v/v) in PBS prior to blocking in 10% normal goat serum (v/v) for 30 min. After overnight incubation with a primary antibody (**Table 1**), Alexa Fluor dye-conjugated secondary antibodies generated in goat (Invitrogen, 1∶200 dilution) were used for protein visualization. Nuclei were stained with DAPI (4′,6-diamidino-2-phenylindole) (Invitrogen). For F-actin staining, frozen cross-sections of mouse testes were fixed in 4% PFA (w/v) in PBS at room temperature for 10 min, permeabilized and subsequently blocked by using 1% BSA (w/v) in PBS for 30 min, followed by an incubation with FITC-conjugated phalloidin (Sigma-Aldrich, 1∶50 dilution) for 30 min. Images were acquired using an Olympus BX61 fluorescence microscope with the Olympus MicroSuite Five software package (Version 1224) in TIFF format. Images were analyzed and overlaid using Photoshop CS3 Extended software (Adobe Systems). To reduce inter-experimental variations, cross-sections from testes of N-WASP^SC-cKO^ and control mice were processed in pairs simultaneously.

### Immunohistochemistry

Immunohistochemistry (IHC) was performed using paraffin-embedded mouse testes as described [49]. In brief, 4-µm thick sections were cut and placed on Superfrost Plus Micro Slides (VWR International). For antigen/epitope retrieval, slides were microwave-heated twice (5 min each) in 10 mM sodium citrate (pH 6.0 at 22°C). Thereafter, sections were immersed sequentially in 3% hydrogen peroxide (v/v) for 20 min and Triton X-100 (0.1%, v/v) and normal goat serum (10%, v/v) in PBS, which were used as penetration enhancer and blocking solution, respectively. After overnight incubation with anti-connexin 43 antibody (**Table 1**), a biotin-conjugated F(ab′)_2_ fragment of goat anti-rabbit IgG and HRP-streptavidin conjugate (Invitrogen) were added to the sections in succession, and a color reaction was obtained using 3-amino-9-ethylcarbazole (AEC, red precipitate, Invitrogen) as the chromogenic substrate.

### Hematoxylin and eosin (H&E) staining

Bouin's solution-fixed 4-µm-thick paraffin sections were used for H&E staining as described [52]. In brief, sections were rehydrated through successive xylene and ethanol, tap and deionized water. Cell nuclei were stained with Hematoxylin 7211 (Richard-Allan Scientific) for 4–5 min to a blue-purple coloration, followed by immersing in Clarifier and Scott's solution for 1 min, respectively. The cytoplasm was then stained using Eosin-Y for 30 sec to obtain a pink-red color. Sections were dehydrated and cleared with 100% ethanol and xylene, sealed in PolyMount (Polysciences), and examined microscopically.

### Statistical analysis

GB-STAT (V7.0, Dynamic Microsystems) was used for statistical analysis. Student's *t*-test was used to compare changes in protein levels between N-WASP^SC-cKO^ and age-matched control mice, in which the relative protein level was arbitrarily set at 1. For immunofluorescence analysis, each time point represented at least *n* = 3 mice. For immunoblotting data, each bar is the mean±SD of 4 mice including control group. *P*<0.05 was interpreted as statistically significant.
